# Age-associated augmented renal clearance and low BMI trigger suboptimal vancomycin trough concentrations in children with haematologic diseases: data of 1453 paediatric patients from 2017 to 2022

**DOI:** 10.1186/s12887-023-04288-4

**Published:** 2023-10-25

**Authors:** Fengjiao Wang, Mi Zhou, Wenjuan Wang, Zengyan Zhu, Yinghui Yan

**Affiliations:** Department of Pharmacy, Children’s Hospital of Soochow University, 92# Street Zhongnan, Suzhou, 215025 Jiangsu China

**Keywords:** Haematologic diseases, Children, Vancomycin, Augmented renal clearance

## Abstract

**Background:**

It is usually difficult for the trough concentration of vancomycin to reach the recommended lower limit of 10 mg/L per the label dose in the paediatric population. Moreover, children with haematologic diseases who suffer from neutropenia are more likely to have lower exposure of vancomycin, and the risk factors have been poorly explored.

**Method:**

We reviewed and analysed the initial trough concentration of vancomycin and synchronous cytometry and biochemical parameters in the blood of 1453 paediatric patients with haematologic diseases over a 6 year period, from 2017 to 2022.

**Results:**

Forty-five percent of the enrolled children had vancomycin trough concentrations below 5 mg/L after receiving a dose of 40 mg/kg/day, and the multiple regression showed that age (OR = 0.881, 95% CI 0.855 to 0.909, *P* < 0.001), BMI (OR = 0.941, 95% CI 0.904 to 0.980, *P* = 0.003) and the glomerular filtration rate (OR = 1.006, 95% CI 1.004 to 1.008, *P* < 0.001) were independent risk factors. A total of 79.7% of the children experienced augmented renal clearance, which was closely correlated to age-associated levels of serum creatinine. The vancomycin trough concentration was higher in children with aplastic anaemia than in those with other haematologic diseases due to a higher BMI and a lower glomerular filtration rate.

**Conclusion:**

Age-associated augmented renal clearance and low BMI values contributed to suboptimal trough concentrations of vancomycin in children with haematologic diseases, and the effects of long-term use of cyclosporine and glucocorticoids need to be taken into account.

**Supplementary Information:**

The online version contains supplementary material available at 10.1186/s12887-023-04288-4.

Patients with haematological diseases, especially haematologic malignancies, have a higher risk of acquiring a bloodstream infection (BSI) and thus a mortality rate of 7.1% to 42% [1]. Since febrile neutropenia resulting from cytotoxic chemotherapy or protopathic diseases is the most common risk factor, the immediate initiation of empirical antibiotic therapy is strongly recommended in such circumstances [2]. Vancomycin is a preferred choice for certain cases, such as haemodynamic instability, pneumonia, and catheter-associated infection [3], and therapeutic drug monitoring (TDM) is necessary to maximize clinical efficacy while minimizing the risk of toxicities.

Measures to ensure the effectiveness and safety of vancomycin in paediatric patients are the main focuses of attention for clinicians and pharmacists. Regardless of disease status, there are different recommended dosages of vancomycin for children, such as an initial dose of at least 60 mg/kg/d for paediatric patients aged 1 month to 18 years [4], a dose of 60–80 mg/kg/d for children aged 3 months to 12 years, and a dose of 60–70 mg/kg/d for children aged > 12 years [5]. However, there are no data from paediatric populations suffering from specific diseases, such as haematological diseases. Stéphanie Leroux et al. demonstrated that data on anti-infectives, including vancomycin, are limited in children with cancer and that their optimal dosing regimen remained controversial or undefined [6].

Recently, the concept of augmented renal clearance (ARC) has emerged, which is described as enhanced renal clearing capacity primarily occurring in critically ill patients [7]. Previous studies have demonstrated that whether standard doses are used, ARC is responsible for the failure of β-lactam antibiotics to meet PK/PD targets [8]. It is worth noting that the pharmacokinetics of vancomycin are also influenced by ARC. Recently, a study indicated that high creatinine clearance (CL) in ARC patients is negatively correlated with vancomycin trough concentration, thus resulting in subtherapeutic vancomycin trough concentration and a low area under the concentration–time curve (AUC) to minimal inhibitory concentration (MIC) ratio [9]. In fact, patients with haematologic malignancies or neutropenia have been reported to have enhanced renal clearance, which would affect the systematic exposure of antibiotics predominantly excreted through urine, including vancomycin [10]. Meanwhile, in critically ill children with ARC, inadequate vancomycin treatment doses may lead to increased infection-related morbidity and mortality [11]. However, the dose and trough concentration levels of vancomycin in children with haematological diseases are unclear, and the role and influencing factors of ARC in this population need to be further clarified. Therefore, our study aims to analyse the influencing factors of suboptimal vancomycin trough concentrations in children with haematological diseases to clarify the role of ARC and its risk factors, thus providing a reference for optimized vancomycin therapy for this population.

## Materials and methods

### Study design

Data were collected from children with haematologic diseases who were hospitalized at the Children’s Hospital of Soochow University from 2017 to 2022. Patients were treated with intravenous vancomycin for a confirmed or suspected gram-positive infection and had at least one vancomycin serum concentration that was assayed for TDM. We enrolled patients who met the following criteria: (i) children between 1 month and 18 years old with haematologic diseases; (ii) children with available data on initial trough concentrations obtained after 4 doses of intravenous vancomycin; and (iii) children with available results of blood biochemistry, including hepatic and renal function, and blood cytometry within 48 h before the day of the initial TDM sample collection. Patients receiving renal replacement therapy were excluded. Although some enrolled children had more than one result of vancomycin trough concentration, only the initial results were included in the data analysis.

### Dosing regimen and sampling

Vancomycin was administered as an intravenous infusion over 60 min. The empirical initial dosing regimen was 40 to 60 mg/kg/day in three or four divided doses, and for older children with a high body weight, the daily dose may also be divided into two doses. Regardless, vancomycin concentrations were monitored 30 min before the fifth or sixth doses of intravenous vancomycin to obtain a steady-state trough concentration.

### Assay of serum vancomycin and eGFR

The serum vancomycin concentrations were determined by fluorescence polarization immunoassay (FPIA) using an automatic biochemical analyser (Viva-E, Siemens, Berlin, Germany). We used a modified Schwartz formula $eGFR=41.3[height/Scr]$ [12] to estimate the glomerular filtration rates on the day of the initial TDM sample collection for each patient.

### Statistics analysis

All statistical analyses were performed using IBM SPSS Statistics 26 (International Business Machines Corporation, Armonk, New York, USA), and figures were generated by GraphPad Prism 8 (GraphPad Software, San Diego, California, USA). For quantitative data, normality analysis was performed through histograms and the Shapiro‒Wilk test, and after passing the normality test, data are represented by means (standard deviation), with comparison by t test or one-way analysis of variance. Quantitative data not conforming to a normal distribution are represented by the median (quartile), with comparisons by the Mann‒Whitney U test, Kruskal‒Wallis test and Kruskal‒Wallis ANOVA test. Categorical variables are represented by the number of cases (constituent ratio), and the comparison between groups was completed through the chi-square test. Pearson or Spearman analysis was selected for correlation tests considering whether variables satisfied a normal distribution. Correlations for all analyses were assessed at a significance level of α = 0.05.

For the establishment of binary and multiple logistic regression models, goodness of fit was tested by the Hosmer Lemeshow test, and the likelihood ratio test was used to screen variables and compare the merits and demerits.

## Results

### Study population and characteristics

**Fig. 1:**
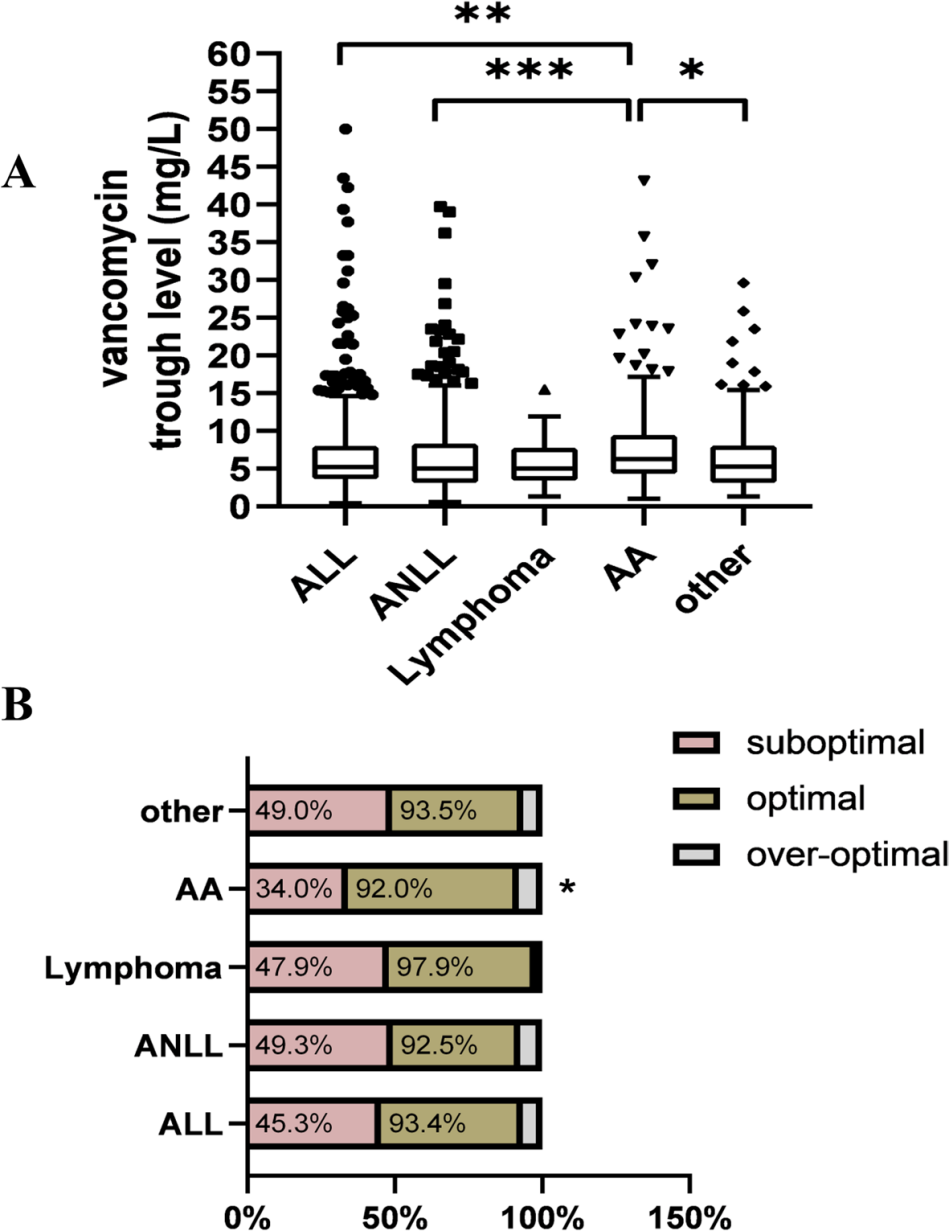
Serum trough concentrations and optimal percentage of vancomycin in children with different haematologic diseases. **A** The median vancomycin trough concentration in children with different haematologic diseases; **B** The proportion of optimal vancomycin serum concentration (5–15 mg/L) in children with different haematologic diseases

A total of 1,453 vancomycin serum concentration tests from 1,453 children were included in this study. The demographic data and clinical characteristics of the paediatric patients are shown in Table 1. The median age of the enrolled children was 6.7 years old, with 9.57% being younger than 2 years old, 73.71% 2 to < 12 years, and 16.72% 12 to < 18 years. The initial dose of vancomycin was approximately 40 mg/kg/d. The median neutrophil count of the enrolled children was 0.1*10^9^/L, and the estimated glomerular filtration rate was 169.85 mL/min/1.73m^2^. The haematologic diseases diagnosed in the enrolled children were principally acute lymphoblastic leukaemia (ALL), acute nonlymphocytic leukaemia (ANLL), which included acute myeloid leukaemia and juvenile monocytic leukaemia, and aplastic anaemia (AA). A small number of lymphomas were listed separately, and the remaining were classified into other categories. A total of 1,453 vancomycin serum concentrations were grouped based on the haematologic disease types, as shown in Fig. 1. The median serum concentrations of vancomycin in children with aplastic anaemia were relatively high, at 6.3 mg/L, compared with ALL (5.2 mg/L, **P = 0.003), ANLL (5.3 mg/L, ***P < 0.001) and other haematologic diseases (5.05 mg/L, *P = 0.011), as shown in Fig. 1A. In addition, the proportion of optimal vancomycin serum concentration (5–15 mg/L) in children with aplastic anaemia was significantly larger than that in the other haematologic disease groups (58% compared with 48.1% in ALL, 43.2% in ANLL, 50% in lymphomas and 44.5% in other, *P < 0.05 in Fig. 1B).

**Table 1:** Clinical characteristics of 1453 patients

	**Value**	
**Characteristics** ^a^	**No. (%)**	**Median (P25, P75) /Average (SD)**
Patients	1453	
Infants (< 2yrs)	139(9.57%)	
Children (2 to 12yrs)	1071(73.71%)	
Adolescents (12 to 18yrs)	243(16.72%)	
Samples	1453	
Sex (male)	867(59.67%)	
Age (yrs)		6.7(3.7, 10.7)
Weight (kg)		21.7(15, 34.5)
BMI (kg/m^2^)		15.91(14.42, 17.88)
ALB (g/L)		38.85(0.11)
ALT (U/L)		25.1(14.6, 45.8)
AST (U/L)		24.6(17, 36.75)
ALP (U/L)		122(92, 163.05)
GTT (U/L)		24.2(15.5, 50.55)
TBIL (µmol/L)		10.7(7.30, 15, 71)
DBIL (µmol/L)		4.24(2.82, 6.63)
IBIL (µmol/L)		6.3(4.32, 9.14)
BUN (mmol/L)		3.69(2.73, 4.85)
SCR (µmol/L)		25.4(19.65, 33.7)
LDH (U/L)		220.6(174, 310.95)
WBC (*10^9/L)		0.75(0.07, 2.47)
NE (*10^9/L)		0.1(0.00, 0.95)
HLB (g/L)		78(69, 89)
PLT (*10^9/L)		44(24, 86)
eGFR		169.85(137.11, 207.31)
Hematologic malignancy type		
Acute lymphoblastic leukemia	594(40.88%)	
Acute non-lymphocytic leukemia	444(30.56%)	
lymphoma	48(3.3%)	
Aplastic anemia	212(14.59%)	
other	155(10.67%)	
Vancomycin dose(mg/kg/day)	39.6(37.5, 42.36)	
Vancomycin trough concentration (mg/L)	5.3(3.5, 8.3)	

^a^ *BMI* Body Mass Index, *ALB* Albumin, *ALT* Alanine transaminase, *AST* Aspartate transferase, *ALP* Alkaline phosphatase, *GTT* Glutamyl transpeptidase, *TBIL* Total bilirubin, *DBIL* Direct bilirubin, *IBIL* Indirect bilirubin, *BUN* Blood urea nitrogen, *SCR* Serum creatinine, *LDH* Lactic dehydrogenase, *WBC* White blood cells, *NE* Neutrophil, *HLB* Hemoglobin, *PLT* Blood platelet, *eGFR* estimate glomerular filtration rate

### Younger age, lower BMI and higher glomerular filtration rate are independent risk factors for suboptimal vancomycin trough concentrations in children with haematologic diseases

To investigate what factors influence the trough concentration of vancomycin in children with haematologic diseases, we classified the enrolled children into three groups: the suboptimal group (serum trough concentration < 5 mg/L), the optimal group (serum trough concentration 5 mg/L to 15 mg/L) and the overoptimal group (serum trough concentration > 15 mg/L), based on the optimal trough concentration in the guidelines for therapeutic drug monitoring of vancomycin in paediatric patients [4]. The suboptimal vancomycin trough concentration was more frequently observed in younger children who had a lower body weight, lower body mass index (BMI), lower liver function index, lower renal function index, lower white blood cell and neutrophil counts, higher platelet counts and higher glomerular filtration rate, as demonstrated in Table S1.

**Fig. 2:**
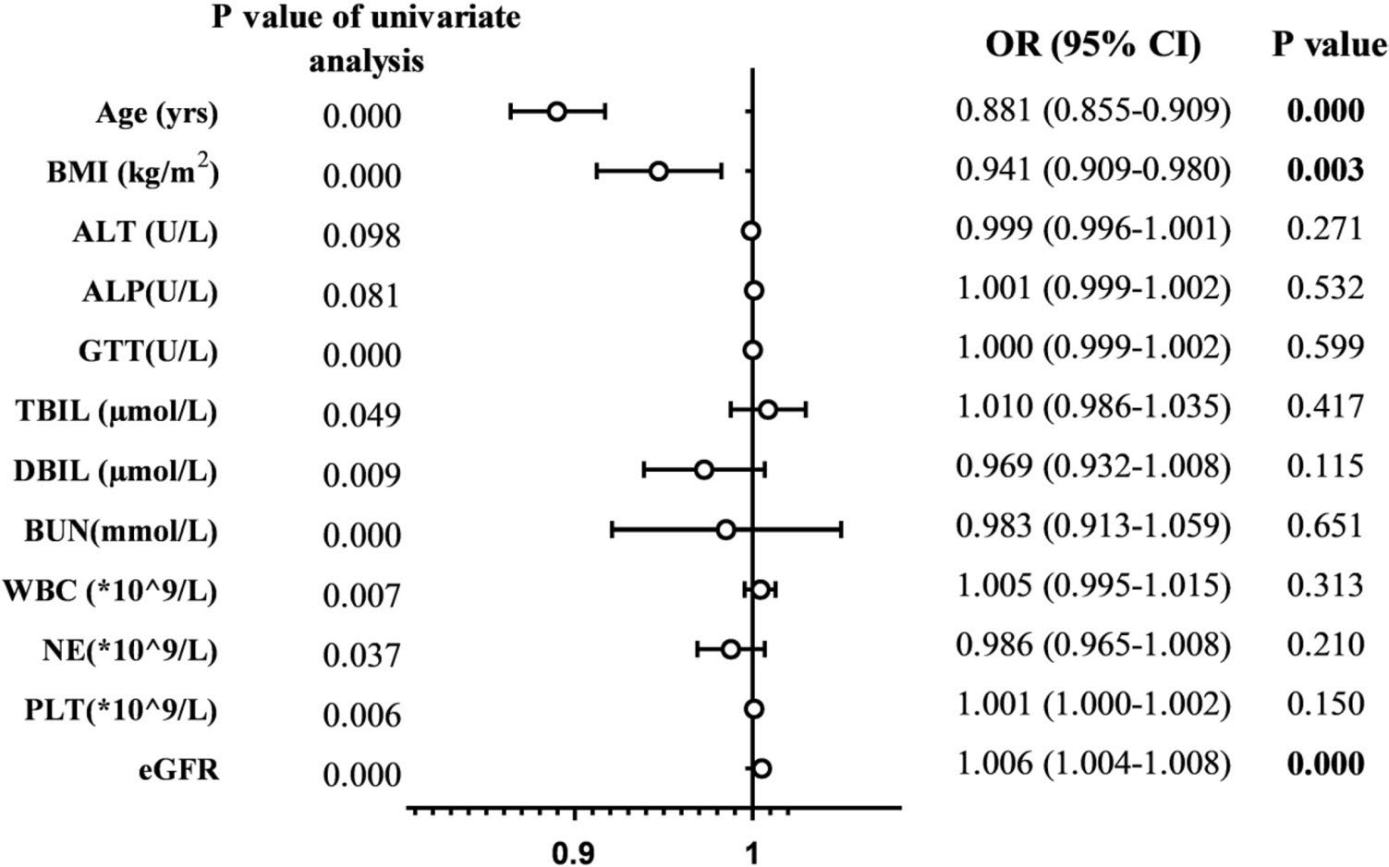
Forest plot for suboptimal vancomycin trough concentration in children with haematologic diseases

To further determine the risk factors related to suboptimal vancomycin trough concentration, the covariates with a *P* value < 0.1 in the univariate analysis were included in the multiple logistic regression analysis in three groups, and the optimal group was taken as a reference. As shown in Fig. 2, significant differences were found in the partial regression coefficients of age (OR = 0.881, 95% confidential interval: 0.855 to 0.909, *P* < 0.001), BMI (OR = 0.941, 95% confidential interval: 0.909 to 0.980, *P* = 0.003) and glomerular filtration rate (OR = 1.006, 95% confidential interval: 1.004 to 1.008, *P* < 0.001). In contrast, the overoptimal group showed completely different results compared to the optimal group, except for the glomerular filtration rate (Table S2). Briefly, these results suggest that younger age, lower BMI and higher glomerular filtration rate are independent risk factors for suboptimal vancomycin trough concentration in children with haematologic diseases.

### Age-associated augmented renal clearance and vancomycin trough concentrations

**Fig. 3:**
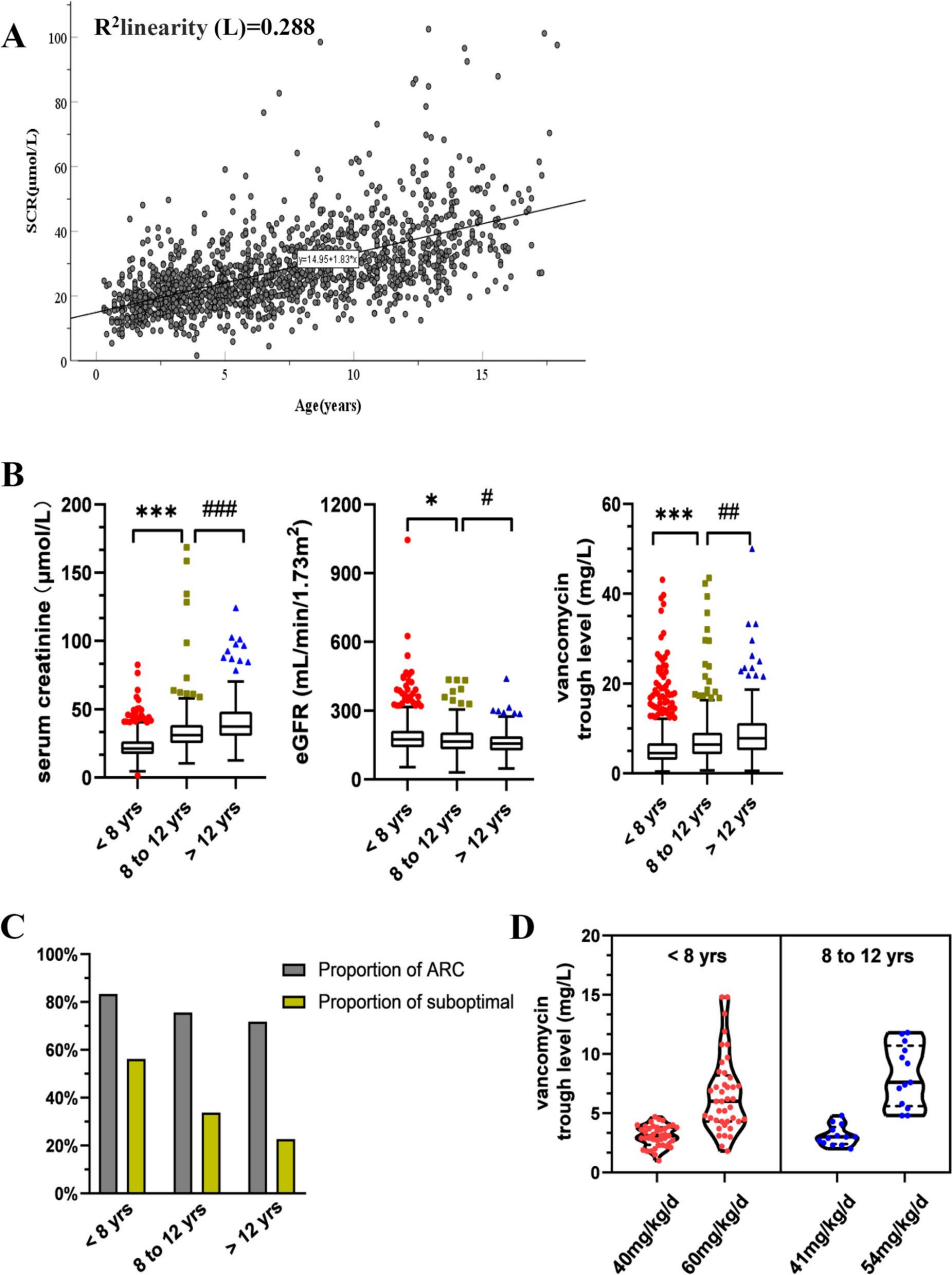
Age-associated augmented renal clearance and vancomycin trough concentrations. **A** Age was positively correlated with serum creatinine levels; **B** Serum creatinine levels, eGFR, and vancomycin trough concentrations stratified by age; **C** Proportion of ARC and suboptimal vancomycin trough concentrations stratified by age; **D** Violin plot for serum trough concentrations of vancomycin after dose adjustment in children of different ages

ARC is a phenomenon of increased renal function in patients with risk factors, including trauma, surgery, sepsis, burn, subarachnoid haemorrhage, and haematological malignancy [13]. Since an increased glomerular filtration rate was demonstrated to be an independent risk factor for suboptimal vancomycin trough concentration in our study, we divided the enrolled children into ARC and non-ARC groups according to the definition of ARC [14] and compared the possible influencing factors between them. We can judge from Table 2 that age, body weight, BMI, blood urea nitrogen, serum creatinine, white blood cell and neutrophil counts, glomerular filtration rate and proportion of neutropenia are significantly different between the ARC and non-ARC groups. However, in the binary logistic regression, age was changed into a grade variable (< 12 years old and > 12 years old) due to no linear relationship with the logit conversion value of the dependent variable, and body weight was excluded due to collinearity with age. The results showed that age, serum creatinine and the interactive effect of age and serum creatinine were statistically significant for ARC (Table S3). There was a monotone correlation between age and serum creatinine level with a Spearman coefficient of 0.62 (***P < 0.001 in Fig. 3A). Strata-specific analyses in Figs. 3B and C demonstrated significant difference in serum creatinine levels (21.3 µmol/L in < 8 years, 31 µmol/L in 8 to 12 years, 37.35 µmol/L in > 12 years, *P* < 0.001) among children of different ages, which appeared likewise in glomerular filtration rates (174.81 mL/min/1.73m^2^ in < 8 years, 165.97 mL/min/1.73m^2^ in 8 to 12 years, 156.62 mL/min/1.73m^2^ in > 12 years, *P* < 0.001 between < 8 years and > 12 years) and the ratio of ARC(83.4% in < 8 years, 75.6% in 8 to 12 years, 71.8% in > 12 years, *P* < 0.001). Furthermore, the difference in renal clearance is also reflected in the drug concentration. The trough concentration of vancomycin (4.5 mg/L in < 8 years, 6.4 mg/L in 8 to 12 years, 7.85 mg/L in > 12 years, *P* < 0.001) and the proportion of suboptimal concentrations in children of various ages (56.2% in < 8 years, 33.8% in 8 to 12 years, 22.7% in > 12 years, *P* < 0.001) showed the corresponding trend.

**Table 2:** Baseline comparison of ARC and non-ARC

**Characteristics** ^a^	**Median (P25, P75) / No. (%)**		**χ** ^**2**^ **/t/Z**	***P *value**
	**ARC**	**Non-ARC**		
Number	1158(79.70%)	295(20.3%)		
Age (yrs)	6.4(3.7, 10.325)	8.4(3.8, 11.3)	-2.826	**0.005**
Sex (male)	678(58.5%)	189(64.1%)	2.975	0.085
Weight (kg)	21(15, 32.7)	25.4(15, 41)	-3.42	**0.001**
BMI (kg/m^2^)	15.73(14.36, 17.47)	16.82(14.87, 19.32)	-5.278	**0.000**
BUN (mmol/L)	3.54(2.64, 4.68)	4.4(3.34, 5.6)	-7.609	**0.000**
SCR (µmol/L)	23.1(18.2, 28.7)	41(33.4, 50.9)	-21.6	**0.000**
WBC(*10^9/L)	0.62(0.05, 2.27)	1.25(0.27, 3.12)	-4.863	**0.000**
NE(*10^9/L)	0.07(0.00, 0.86)	0.19(0.01, 1.14)	-4.05	**0.000**
Neutropenia	807(69.7%)	186(63.1%)	4.788	**0.029**
Sepsis	948(81.9%)	244(82.7%)	0.114	0.735
eGFR	182.259(157.128, 217.942)	111.948(98.145, 121.617)	-26.549	**0.000**

^a^ *BMI* Body Mass Index, *BUN* Blood urea nitrogen, *SCR* Serum creatinine, *WBC* White blood cells, *NE *Neutrophil, *eGFR* Estimate glomerular filtration rate

In our study, some of the children whose vancomycin trough concentrations were suboptimal had their dose adjusted, and therapeutic drug monitoring was performed later, as shown in Fig. 3D. The average dose of vancomycin in 41 children < 8 years was increased from 40 mg/kg/d to 60 mg/kg/d, and 39% of the samples were still suboptimal, with the median trough concentration increasing from 3.2 mg/L to 6.2 mg/L. In contrast, the average dose of 13 children between 8–12 years was increased from 41 mg/kg/d to 54 mg/kg/d, and the median trough concentration was increased from 3.1 mg/L to 7.4 mg/L, with 15% suboptimal.

### Children with aplastic anaemia had higher vancomycin trough concentrations owing to a higher BMI and a lower glomerular filtration rate

Based on the results above, we compared age, BMI, glomerular filtration rate and other characteristics between children with different haematologic diseases. The results shown in Table S4 revealed that there was no difference in age between the aplastic anaemia group and the other three groups. However, children with aplastic anaemia were observed to have a significantly higher BMI, lower glomerular filtration rate and lower proportion of ARC when compared with the other four groups.

## Discussion

Our study revealed the independent risk factors affecting suboptimal vancomycin trough concentration in children with haematologic diseases and confirmed that augmented renal clearance was a crucial part. Thanks to a large sample size, we were able to identify that age-mediated serum creatinine level was the major factor associated with ARC, and age-associated ARC played a dominant role in suboptimal vancomycin trough concentration in children with haematologic diseases.

The clinical application of vancomycin in children has always attracted attention. In recent years, more studies have focused on the pharmacokinetics and dose optimization of vancomycin in paediatric populations, and the results confirmed that in neonates and infants [15] and children with varying levels of obesity and renal dysfunction [16], the pharmacokinetic models of vancomycin are totally different. In addition, it is difficult to achieve the trough concentration of vancomycin recommended for adults (10–15 mg/L) [4] in children, and some studies [5, 17] have shown that trough concentrations of 5–10 mg/L can also reach a AUC/MIC ratio > 400 and were not associated with increased 30-day mortality or recurrent bacteraemia when compared with concentrations > 10 mg/L [18] in paediatric patients, even though the target is initially for the treatment of methicillin-resistant Staphylococcus aureus infections in adults and children [19]. Therefore, according to the latest guidelines, steady-state trough concentrations of vancomycin are recommended to be maintained at 5–15 mg/L in paediatric patients or neonates [4].

Haematologic disease, especially haematologic malignancy is common in paediatric patients who are treated with vancomycin. Izumisawa et al. [20] found that adult patients with haematologic malignancies had significantly higher vancomycin clearance and lower serum concentrations of vancomycin than patients without malignancies. In the few studies focusing on pharmacokinetics in paediatric patients with haematologic malignancies [6], weight was always significant, whereas creatinine clearance was significant only in the model by Zhao [21]. However, in our study, where most of paediatric patients were haematologic malignancies, the independent risk factors for suboptimal vancomycin were age, BMI and glomerular filtration rate. Notably, in children, there is a strong correlation between body weight and age, but BMI can be used to objectively evaluate the body mass of children. Ultimately, younger age, increased glomerular filtration rate and lower BMI, which are rarely mentioned, could lead to suboptimal vancomycin concentrations. In adult patients, a high BMI suggests obesity and is thought to increase the volume of distribution [22] and lead to a decrease in vancomycin concentration, whereas in paediatric patients with haematologic diseases in our study, the BMIs ranged from normal to underweight. Vancomycin is a large glycopeptide molecule that is hydrophilic, indicating its decreased distribution into tissues with high lipid concentrations, such as adipose tissue. Heble et al reported higher vancomycin trough concentrations in overweight and obese children than in normal-weight children, with dosing based on total body weight [23]. Marissa et al demonstrated that the increase in BMI in children with acute lymphoblastic leukaemia during the first 32 weeks of treatment was explained by an increase in fat percentage using dual-energy X-ray absorptiometry [24]. Therefore, with the increase in BMI in children with a haematologic disease, despite not being at the level of obesity, the fat percentage was likely to increase, which resulted in a lower distribution and increased trough concentration of vancomycin, making it easier to reach the optimal concentration.

Published data on ARC appear to be limited to adult patients, whereas in recent years, studies have indicated that accelerated clearance of vancomycin exists in children with febrile neutropenia [25] or sepsis [26], which may be related to ARC. Studies in adults with a haematologic malignancy showed that febrile neutropenia is associated with low vancomycin concentrations, possibly driven by ARC [27]. Our study in paediatric patients with haematologic diseases demonstrated a high prevalence of ARC and that neutropenia was a possible influencing factor in the univariate analysis, but serum creatinine level mediated by age was the dominant factor for ARC in the multivariate regression. Few studies evaluated the risk factors for ARC in paediatric patients, which involved age above 7.9 years, low serum creatinine, male sex, and febrile neutropenia [28]. Our data revealed a positive correlation between age and serum creatinine, and this interactive effect was the main risk factor for ARC in children with haematologic diseases, further resulting in a low vancomycin trough concentration. The analysis above suggested that younger age, especially less than 8 years, was associated with a higher risk of ARC and suboptimal vancomycin concentrations among children with haematologic diseases, in which the majority had neutropenia.

In patients with different haematologic diseases, differences in trough concentrations of vancomycin have not been reported. Interestingly, in our study, a significantly higher vancomycin trough concentration and higher proportion of optimal concentration were found in children with aplastic anaemia, and aplastic anaemia is not a haematologic malignancy compared with ALL, ANLL and lymphoma. The analysis of risk factors contributed to finding significant differences between BMI and eGFR in children with different diseases. We assume that the unique treatment regimen for aplastic anaemia [29] (IST with horse anti-thymocyte globulin (ATG) and cyclosporine therapy), including the prolonged use of cyclosporine and four weeks of glucocorticoid therapy [30], is responsible for the significant difference in eGFR and BMI in children with aplastic anaemia, resulting in a higher trough concentration of vancomycin.

In our study, children of different ages with a haematologic disease had different trough concentrations after the dose of vancomycin was adjusted. Especially for children younger than 8 years old, nearly half of the samples did not have a trough concentration of 5 mg/L at a dose of 60 mg/kg/d. These data also suggest that 60 mg/kg/d vancomycin may not be sufficient for children with haematologic diseases and who are younger than 8 years old, and it is urgent to establish a population pharmacokinetic model of vancomycin to explore the optimal dose.

## Conclusion

Based on the database of children with haematologic diseases who received intravenous vancomycin, we demonstrated that age-associated augmented renal clearance and low BMI values contributed to the suboptimal trough concentration of vancomycin in this population. Especially for children younger than 8 years old, a dose of 60 mg/kg/day was insufficient to achieve a trough concentration of 5 mg/L. In addition, children with aplastic anaemia, who have been using cyclosporine and glucocorticoids for a longer time, are more likely to show the optimal vancomycin trough concentration than those with haematologic malignancies.

### Supplementary Information

**Additional file 1: Table S1.** Baseline comparison of different vancomycin trough concentration levels. **Table S2.** Multivariate regression model for over-optimal vancomycin trough concentration. **Table S3.** Multivariate regression model for ARC. **Table S4.** Comparison of risk factors among children with different hematologic malignancy.

## Data Availability

The datasets used and/or analysed during the current study are available from the corresponding author upon reasonable request.

## Abbreviations

BMI: Body Mass Index
BSI: Blood Stream Infection
TDM: Therapeutic Drug Monitoring
ARC: Augmented Renal Clearance
CL: Creatinine clearance
AUC: Area Under the concentration–time Curve
MIC: Minimal Inhibitory Concentration
eGFR: Estimate Glomerular Filtration Rate
ALL: Acute Lymphoblastic Leukemia,
ANLL: Acute Non-Lymphocytic Leukemia
AA: Aplastic Anemia
IST: Immunosuppressive therapy
