# Supplementary material for: Age-associated augmented renal clearance and low BMI trigger suboptimal vancomycin trough concentrations in children with haematologic diseases: data of 1453 paediatric patients from 2017 to 2022
Source: BMC Pediatr. 2023 Oct 25;23:528. doi: 10.1186/s12887-023-04288-4 (PMC10601245; doi:10.1186/s12887-023-04288-4)
Supplement: Supplementary file 1 — Additional file 1: Table S1. Baseline comparison of different vancomycin trough concentration levels. Table S2. Multivariate regression model for over-optimal vancomycin trough concentration. Table S3. Multivariate regression model for ARC. Table S4. Comparison of risk factors among children with different hematologic malignancy. [file 12887_2023_4288_MOESM1_ESM.pdf]

TABLE S1 Baseline comparison of different vancomycin trough concentration levels

Characteristics[a]	Median (P25, P75) /Average (SD)/No. (%)			χ^2/F/H	P value
	Suboptimal (<5 mg/L)	Optimal (5 to 15 mg/L)	Over-optimal (>15 mg/L)		
Number	659(45.35%)	694(47.76%)	100(6.88%)		
Vancomycin trough concentration (mg/L)	3.3(2.5, 4.1)	7.3(6, 9.6)	18.65(16.425, 24.825)		
Vancomycin dose(mg/kg/day)	39.47(37.71, 41.51)	39.62(37.5, 42.86)	40(38.1, 44.18)	2.388	0.303
Age (yrs)	5.1(3, 8.3)	8.4(4.8, 11.8)	9.65(4.475, 12.2)	111.522	**0.000**
Sex (male)	382(57.97%)	421(60.66%)	64(64%)	1.858	0.395
Weight (kg)	18.5(14, 26.2)	26(17.5, 42)	29.5(17.35, 42.12)	125.426	**0.000**
BMI (kg/m2)	15.51(14.23, 16.98)	16.37(14.65, 18.70)	16.44(14.17, 18.95)	41.426	**0.000**
ALB (g/L)	38.9(0.17)	38.89(0.16)	38.17(0.42)	1.25	0.287
ALT (U/L)	24.2(14.4, 41.2)	25.9(14.9, 52.6)	25.75(14.77, 52.05)	4.648	0.098
AST (U/L)	24(16.8, 35.8)	25.1(17.07, 38.1)	24.3(18.4, 33.35)	1.192	0.551
ALP (U/L)	125(96, 165)	120(89, 162.25)	114(83, 161.95)	5.018	0.081
GTT (U/L)	21.7(14.8, 41)	27.6(16.1, 58.52)	28.4(16.12, 80.2)	21.272	**0.000**
TBIL (μmol/L)	10.3(7.1, 15.5)	11.4(7.68, 15.82)	10.55(7.15, 15.77)	6.019	**0.049**
DBIL (μmol/L)	3.93(2.7, 6.4)	4.59(3.02, 6.73)	4.35(2.93, 6.7)	9.437	**0.009**
IBIL (μmol/L)	6.2(4.2, 9.12)	4.5(3.11, 6.46)	6.31(4, 8.4)	3.029	0.220
BUN (mmol/L)	3.62(2.64, 4.75)	3.68(2.76, 4.84)	4.47(3.23, 6.05)	23.014	**0.000**
SCR (μmol/L)	21.8(17.3, 27.6)	28.6(22.1, 37.12)	36.1(25.00, 52.5)	207.655	**0.000**
LDH (U/L)	218.8(173.7, 308.3)	224.15(176.9, 317.02)	220.6(170.32, 324.47)	1.472	0.479
WBC(*10^9/L)	0.72(0.06, 2.34)	0.68(0.07, 2.44)	1.36(0.22, 3.58)	10.026	**0.007**
NE(*10^9/L)	0.1(0.00, 0.91)	0.09(0.00, 0.88)	0.19(0.02, 1.48)	6.589	**0.037**
HLB (g/L)	76(68, 89)	78(69, 89)	80.5(71, 93)	3.729	0.155
PLT (*10^9/L)	50(27, 93)	40(23, 78)	42.5(25, 88.25)	10.072	**0.006**
eGFR	183.62(152.07, 225.66)	159.20(131.38, 191.25)	129.80(95.45, 173.71)	122.23	**0.000**

[a] BMI, body Mass Index; ALB, albumin; ALT, alanine transaminase; AST, aspartate transferase; ALP, alkaline phosphatase; GTT, glutamyl transpeptidase; TBIL, total bilirubin; DBIL, direct bilirubin; IBIL, indirect bilirubin; BUN, blood urea nitrogen; SCR, serum creatinine; LDH, lactic dehydrogenase; WBC, white blood cells; NE, neutrophil; HLB, hemoglobin; PLT, blood platelet; eGFR, estimate glomerular filtration rate.

TABLE S2 Multivariate regression model for over-optimal vancomycin trough concentration

Variates[a]	Wald	SE	OR(95%CI)	P value
Age	0.058	0.028	1.007(0.953-1.064)	0.810
BMI	0.803	0.033	0.971(0.909-1.036)	0.370
ALT	1.589	0.002	0.997(0.993-1.002)	0.207
ALP	6.548	0.002	0.995(0.991-0.999)	**0.011**
GTT	10.308	0.001	1.003(1.001-1.006)	**0.001**
TBIL	0.447	0.025	0.984(0.937-1.032)	0.504
DBIL	0.026	0.035	1.006(0.939-1.077)	0.872
BUN	12.193	0.057	1.222(1.092-1.368)	**0.000**
WBC	0.000	0.010	1.000(0.980-1.020)	0.997
NE	0.128	0.019	0.993(0.956-1.032)	0.721
PLT	0.011	0.001	1.000(0.997-1.003)	0.916
eGFR	15.905	0.003	0.989(0.984-0.995)	**0.000**

[a] BMI, body Mass Index; ALT, alanine transaminase; ALP, alkaline phosphatase; GTT, glutamyl transpeptidase; TBIL, total bilirubin; DBIL, direct bilirubin; BUN, blood urea nitrogen; WBC, white blood cells; NE, neutrophil; PLT, blood platelet; eGFR, estimate glomerular filtration rate.

TABLE S3 Multivariate regression model for ARC

Variates[a]	Wald	SE	OR(95%CI)	P value
Age12	11.528	3.269	66131.21(109.08-40094230.03)	**0.001**
SCR	226.602	0.017	0.779(0.754-0.805)	**0.000**
Age12*SCR	5.996	0.072	0.837(0.727-0.965)	**0.014**
BMI	0.226	0.031	0.985(0.928-1.047)	0.635
BUN	3.487	0.061	0.893(0.793-1.006)	0.062
WBC	1.230	0.006	0.993 (0.982-1.005)	0.267
NE	0.091	0.016	0.995(0.965-1.027)	0.762
Neutropenia	0.802	0.220	1.217(0.791-1.872)	0.371

[a] SCR, serum creatinine; BMI, body Mass Index; BUN, blood urea nitrogen; WBC, white blood cells; NE, neutrophil count.

TABLE S4 Comparison of risk factors among children with different hematologic malignancy

	ALL	ANLL	Lymphomas	AA	Other	P value
Age (yrs)	6.5(3.7,10.9)	7.05(3.2,10.8)	8.1(5.53,11.5)	7.55(4.7,10.65)	5.8(2.7,9)***[a]	<0.001
BMI (kg/m2)	15.64(14.05,17.44)***	15.97(14.6,17.99)	15.03(13.67,16.59)***	**16.79(14.83,19.20)**	16.07(14.90,17.85)	<0.001
eGFR	174.67(142.31,209.2)***	171.53(137.62,209.38)***	160.4(123.1,215.0)	**149.79(122.02,184.46)**	175.16(144.34, 212.94)***	<0.001
Proportion of ARC	488(82.2%)	358(80.6%)	33(68.8%)	**146(68.9%)**	132(85.2%)	<0.001

[a] Kruskal-Wallis test intergroup comparison ***P<0.001 compared with aplastic anemia group.
